# Thyroid-like follicular carcinoma of the kidney presenting in a 13-year-old female: a case report from Syria

**DOI:** 10.1093/jscr/rjac467

**Published:** 2022-10-31

**Authors:** Ahmad Al Mousa, Sami Rahmeh, Vairy Rezkallah, Lina Ghabreau, Ibrahim Alhadid

**Affiliations:** Department of Urology, Aleppo University Hospital, Aleppo, Syria; Department of Gastroenterology, Aleppo University Hospital, Aleppo, Syria; Department of Pathology, Aleppo University Hospital, Aleppo, Syria; Department of Pathology, Aleppo University Hospital, Aleppo, Syria; Department of Urology, Aleppo University Hospital, Aleppo, Syria

## Abstract

Thyroid-like follicular carcinoma of the kidney (TLFCK) is a rare cancer that emerged to the medical literature only a few years ago. We present here the first case of thyroid follicular carcinoma-like renal tumor from Syria. The case presented symptomatically and was managed in our surgical unit. Generally, the presenting age described for the previous cases was 19–60 (mean 39.5) with only three cases with younger ages. Here in our case, the patient is only 13 years old making this only the fourth case worldwide of TLFCK in a child. The microscopic view of the tumor showed distinct thyroid features. In addition the immunohistochemical stains played the definitive role in the diagnosis. The staining for Vimentin, and CK19 were diffusively positive. CK7 was focally positive, whereas TTf1, Thyroglobulin, WT1, CD10 and EMA were negative. It is important to consider this diagnosis to spare the patient the treatment protocol of primary thyroid cancer with metastasis.

## INTRODUCTION

Thyroid-like follicular carcinoma of the kidney (TLFCK) is a rare variant of primary renal tumor and was first reported in 2006 [1], it is a subtype of renal cell carcinoma that closely resembles the well-differentiated thyroid follicular neoplasms.

The WHO kidney tumor classification 2016 included it as a variant of renal cell carcinoma and named it thyroid-like follicular renal cell carcinoma [2].

Before a diagnosis is made, pathology has to exclude follicular thyroid cancer metastases to the kidney through immunohistochemical staining.

## CASE PRESENTATION

The patient was a 13-year-old Syrian girl, who presented with a history of right flank pain for 2 months to the Department of Urology in our hospital. There was no history of hematuria, dysuria or thyroid disease or renal disease. On physical examination, her abdomen was smooth without any tenderness or flank masses. The blood tests were normal, and there was no kidney failure, HGB: 11.1 g/dl, Creatinine: 0.61 mg/dl, blood group O negative, her BMI = 19.8 kg/m^2^, slightly increased blood pressure, no weight loss and the rest of the examination was unremarkable.

**Figure 1:**
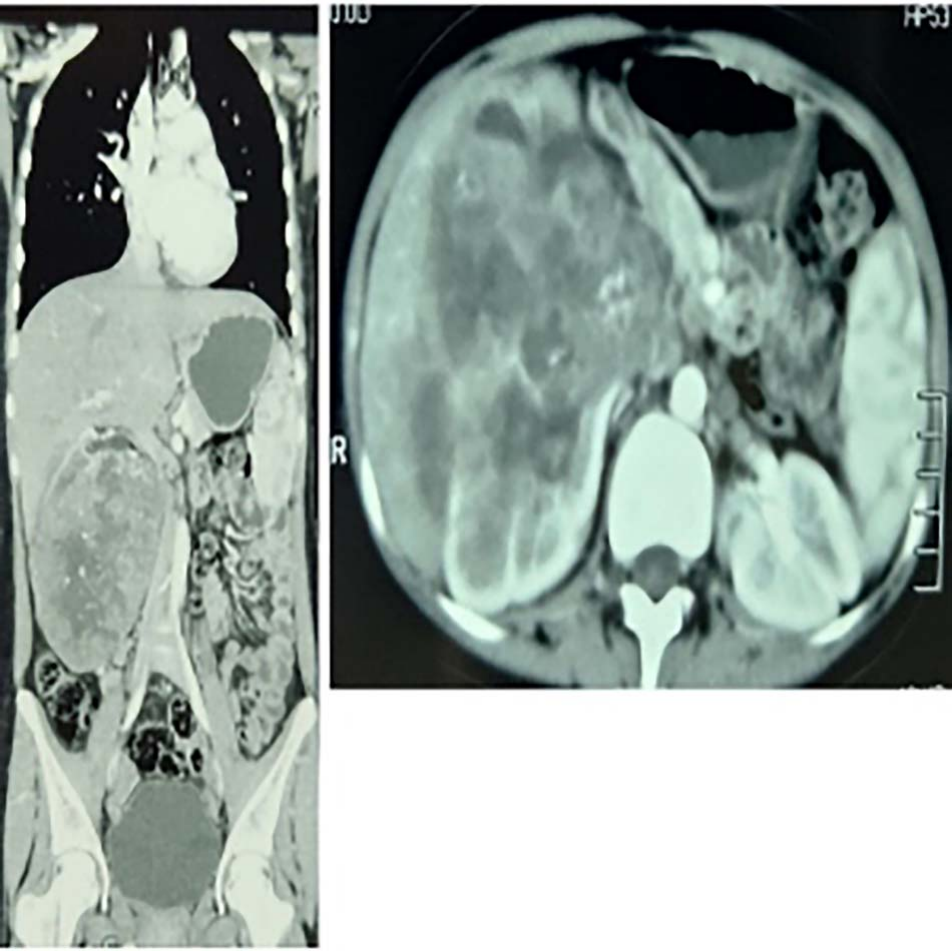
To the left: coronal section multiple-slice computed tomography slice showing the lump at the greatest diameter, to the right a transverse section showing a renal mass.

On imaging, the abdominal ultrasound revealed a huge heterogeneous mass of size 13 × 14 cm encompassing the right kidney. MSCT with contrast of the chest, abdomen and pelvis showed a large right centrally necrotized tumor, homogenously enhancing the contrast material. No distant thoracic metastases, periaortic, inferior vena cava or visceral lymph nodes enlargement, no ovarian mass. There were many stones that filled the left kidney (Fig. 1).

Right radical nephrectomy then was performed through the anterior subcostal incision and no other treatment was arranged and follow-up after 3 months with MSCT of the abdomen and pelvis was clear with no metastasis, and we set-up a longer follow-up program for her.

### Pathology report

**Figure 2:**
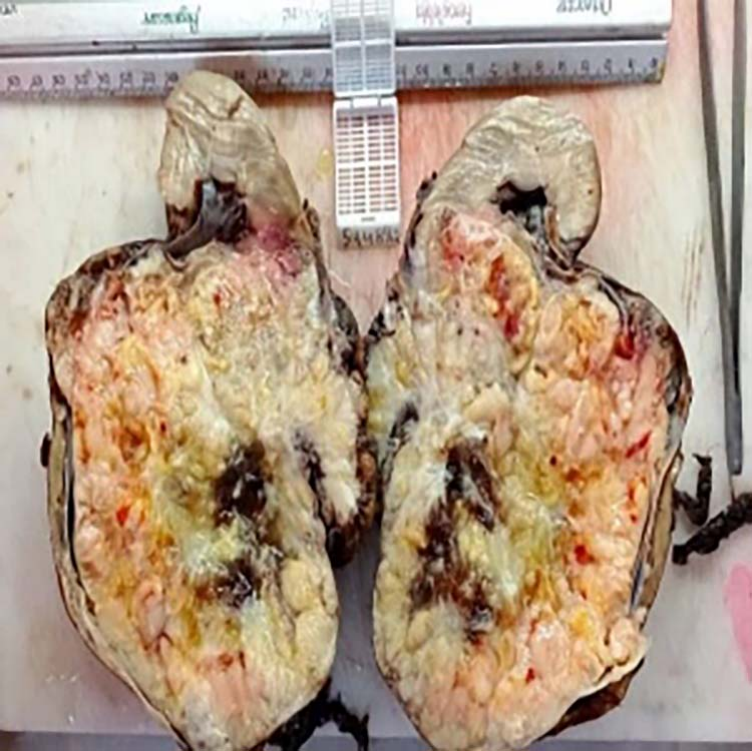
Macroscopic view of the tumor.

After the right nephrectomy, the cut section showed tumor mass located in the lower lobe measures (13.5 × 12.5) cm (Fig. 2).

Histopathology examination revealed follicular structures that consisted of macro and micro follicles filled with eosinophilic amorphous colloid-like material. These follicles are lined by cuboidal cells with finely granular cytoplasm and round conspicuous nuclei. Mitoses are absent and no presence of capsule invasion.

**Figure 3:**
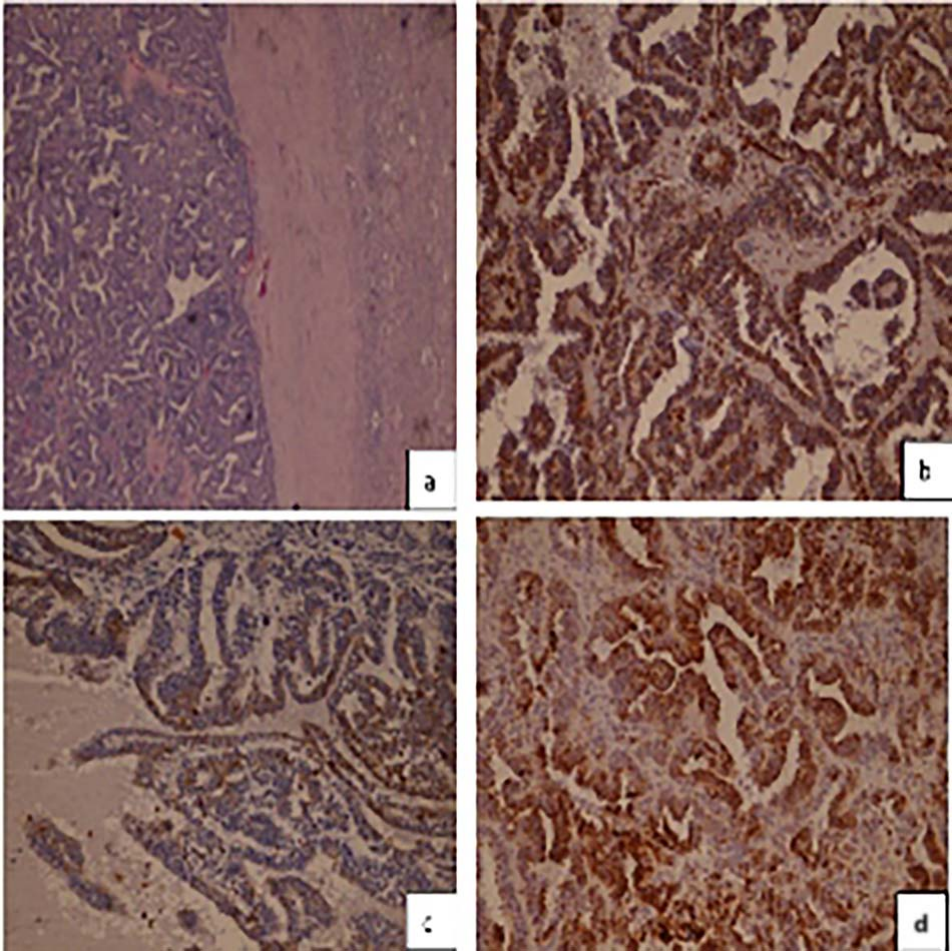
**(a)** Hematoxylineosin staining, (**b**) diffuse positive Vimentin staining (**c**) focally positive CK7 staining (**d**) CK19 positive staining.

Immunohistochemical stains: tumor cells showed intensive staining for Vementin. CK19 were diffusively positive. CK7 was focally positive whilst TTf1, Thyroglobulin, WT1, CD10 and EMA were all Negative (Fig. 3).

## DISCUSSION

At this age, a kidney mass made us suspect Wilms tumor but a biopsy is essential before diagnosis is made.

Follicular morphology and thyroid-like histology leads to considering options other than primary kidney tumor, even though metastasis to the kidney from primary thyroid tumor is very rare and reported only a few times [3], also thyroid clinical examination and Echo Doppler of the neck were in order, looking for nodules or disorders in the thyroid gland, where everything was found normal in our case.

The exclusion of an ovarian mass is important to rule out the possibility of ovarian goiter with metastasis which is again a very rare case to occur [4] but in the frame of the exceptional circumstances of our case, we needed to exclude such possibility.

Clinical review shows that more females are affected by this variant as compared with males (27/12) cases [5] and our case harmonize with this predominance.

Renal primary tumors like clear cell, papillary, or cystic renal cell carcinoma does not habitually show a predominance of follicle-like structures or eosinophilic colloid-like material that resembles thyroid follicular carcinoma [5].

TLFCK tumors are low-grade malignancies, with infrequent metastasis (only six cases were reported; [6]), minimal recurrence (only one case registered) and remission after surgery is the common scenario [5], which means, in general, they have a good prognosis.

Even though some studies claimed that partial nephrectomy could give the same results [7] the size of the tumor at presentation obliged us to do a radical nephrectomy. Follow-up program was set for our patient because the biological behavior of this tumor is not yet fully understood. The first follow up performed to our patient was clear.

Although rare, this entity should be included in the differential diagnosis of renal mass and immunohistochemistry staining should be done. With a growing number of cases described, it has been officially added to the classification of renal cell carcinomas as an emerging entity.

## CONCLUSION

At the moment, the understanding of these tumors is not enough and its biological behavior should be under extensive study to provide physicians with a clear course of action toward such tumor. In addition, immunohistochemical staining is important to be performed in histological diagnosing to type the tumor beyond the morphologic features.
